# Supplementary material for: The Ultra-Processed Food Content of School Meals and Packed Lunches in the United Kingdom
Source: Nutrients. 2022 Jul 20;14(14):2961. doi: 10.3390/nu14142961 (PMC9318725; doi:10.3390/nu14142961)
Supplement: Supplementary file 1 [file nutrients-14-02961-s001.zip › nutrients-1788157-supplementary.pdf]

Supplementary Files

List of Supplementary Tables

Supplementary Table S1- Median ultra-processed food intake (% kcal) at school lunchtime by study covariates and stratified by school phase 2

Supplementary Table S2 - Median ultra-processed food intake (% g) at school lunchtime by study covariates and stratified by school phase 3

Supplementary Table S3 - Logistic regression of the likelihood of consuming minimally and ultra-processed food groups by meal type and school phase (School meals vs reference group packed lunches) 5

Supplementary Table S4 - Quantile (median) regression on ultra-processed food with an interaction between meal type and income group, stratified by school phase 6

Supplementary Table S5 - Sensitivity analysis (1) Additional adjustments for energy, lunch portion and BMI to the quantile regression on ultra-processed food and meal type. 7

Supplementary Table S6 - Sensitivity analysis (2) Quantile regression on ultra-processed food and meal type with exclusion of sample who meal type was not recorded in the dietary diary 7

Supplementary Table S7 - Sensitivity analysis (3) Quantile regression on ultra-processed food and meal type with exclusion of participants who were estimated to have misreported their energy intake 8

List of Supplementary Figures

Supplementary Figure S1 Median intake of UPF by school meals stratified by income and school phase. 4

Supplementary Table S1- Median ultra-processed food intake (% kcal) at school lunchtime by study covariates and stratified by school phase

Variable	Primary		Secondary		Total	
	Median (Q25, Q75)	P[1]	Median (Q25, Q75)	P[1]	Median (Q25, Q75)	P2
	72.6 (55.1,85.7)		77.8 (57.8,95.2)		74.4 (56.2,89.3)	<0.01
**Sex**		0.2		0.03		0.01
Male	73.7 (57.5,87.1)		80.1 (61.4,97.4)		76.2 (58.9,90.4)	
Female	71.7 (51.2,84.4)		75.4 (55.5,93.7)		72.7 (53.1,88.1)	
**Ethnicity**		<0.01		0.39		0.01
White	73.4 (56.8,86.7)		78.6 (59.3,95.6)		75.2 (57.6,90.2)	
Ethnic minorities	66.7 (46.2,82.9)		74.4 (49.2,89.1)		70.6 (47.6,85.3)	
**Income**		0.05		<0.01		<0.01
Low	74.8 (57.8,86.2)		81.6 (63.5,99.6)		77.4 (60.2,92.2)	
Mid	73.3 (55.6,87.4)		78.7 (57.5,95.5)		75.5 (56.2,89.6)	
High	70.3 (51.9,82.8)		71.2 (52,88.3)		70.8 (52,85.2)	
**IMD**		0.03		0.07		0.02
1 (Least deprived)	71 (53.4,85.6)		72.3 (54.8,89.1)		71.4 (53.9,87.6)	
2	73.7 (54.4,84.7)		76.6 (58.9,94.9)		74.9 (56.1,88.9)	
3	71.9 (55.2,83.8)		82.5 (63.2,96.5)		74.3 (57.7,88.3)	
4	75.9 (59.4,88.5)		79.9 (59.7,95.5)		77.1 (59.7,91.3)	
5 (Most deprived)	70.5 (52.9,85.5)		78.6 (55.9,100)		72.9 (54.3,90.1)	
**Region**		0.22		0.91		0.91
England: North	75.4 (59.3,88.4)		77.1 (61.9,93.2)		76.2 (60,89.5)	
England: Central/Midlands	74.8 (57.3,86.7)		76.8 (59,100)		75.9 (58,92.5)	
England: South (incl. London)	70.8 (50.9,83.2)		78.8 (55.5,95)		73 (52.2,88.2)	
Scotland	71.8 (51.8,86.5)		80.1 (61.4,95.2)		73.4 (56.1,88.6)	
Wales	73 (55,86.8)		76.9 (56.7,95)		74.7 (55.9,92.1)	
Northern Ireland	73.5 (59.8,84.3)		79.1 (58.8,96.4)		76.3 (59.4,89)	
**School lunch meal type**		<0.01		<0.01		<0.01
School meal	61 (43.7,75.3)		70.1 (47.7,88.9)		64 (45.3,80.3)	
Packed lunch	81.2 (70.5,91.3)		83.5 (64.8,99.1)		82.1 (67.9,93.9)	

[1]Survey adjusted Kruskal-Wallis test comparing across covariate categories

[2]Survey adjusted Kruskal-Wallis test comparing across primary and secondary schoolchildren

Supplementary Table S2 - Median ultra-processed food intake (% g) at school lunchtime by study covariates and stratified by school phase

Variable	Primary		Secondary		Total	
	Median (Q25, Q75)	P[1]	Median (Q25, Q75)	P[1]	Median (Q25, Q75)	P[2]
	43.7 (28.371)		52.5 (25.485.4)		46.8 (26.977.7)	<0.01
**Sex**		0.07		0.03		0.01
Male	45.4 (29.4,72.4)		58.1 (27.7,87.8)		49.4 (29.3,80.4)	
Female	41.9 (26.9,70)		49.1 (23.9,81.7)		43.2 (25.4,75.4)	
**Ethnicity**		<0.01		0.02		<0.01
White	45.2 (29.1,73.8)		56 (25.7,87.8)		48.6 (27.7,80.6)	
Ethnic minorities	36.6 (25.2,59.1)		43.8 (22.7,70.8)		39.1 (23.9,64.6)	
**Income**		<0.01		0.03		<0.01
Low	47 (31.7,76.7)		58.6 (28.7,93.6)		51.7 (30.5,81.6)	
Mid	46.2 (28.5,76.1)		51.6 (25.4,82.7)		48.1 (27,79.4)	
High	37.9 (25.2,58.7)		41.7 (21.8,76.9)		39.2 (24.1,66.3)	
**IMD**		0.08		0.46		0.44
1 (Least deprived)	40.8 (27.2,61.7)		49.2 (25.9,74.1)		43.7 (26.3,71.5)	
2	40.4 (24.9,72.5)		52.1 (20.9,85.4)		43.5 (22.8,81.2)	
3	42.9 (29.2,67.7)		60.8 (27.9,94.1)		46.1 (28.9,78.6)	
4	49.1 (30.2,76.4)		47.9 (23.6,86)		49.2 (26.9,79.9)	
5 (Most deprived)	43 (29.2,71.9)		58.6 (27,85.4)		48.5 (29,77.3)	
**Region**		<0.01		0.15		<0.01
England: North	46.8 (30.2,73.9)		60.5 (27.9,85.8)		50.9 (29.7,80)	
England: Central/Midlands	51.9 (31.1,78.2)		55.6 (27.5,89.2)		53.6 (30.2,82.1)	
England: South (incl. London)	38.4 (25.2,60.2)		47.3 (23.5,82.7)		40.8 (24.9,70)	
Scotland	51 (29,80.6)		62.8 (23.4,86.8)		53 (28.3,82.7)	
Wales	52.3 (35.6,79.4)		52.9 (25.5,89.6)		52.7 (30.4,83)	
Northern Ireland	42.7 (29.2,70)		47.4 (22.2,81.3)		43.6 (25.9,73.1)	
**School lunch meal type**		<0.01		0.01		<0.01
School meal	35.4 (23.1,48.7)		45.8 (21.9,80.3)		37.7 (22.9,60.2)	
Packed lunch	59.9 (35.6,82.5)		58.6 (29.2,91.2)		59.7 (33.2,86)	

[1] Survey adjusted Kruskal-Wallis test comparing across covariate categories
[2] Survey adjusted Kruskal-Wallis test comparing across primary and secondary schoolchildren

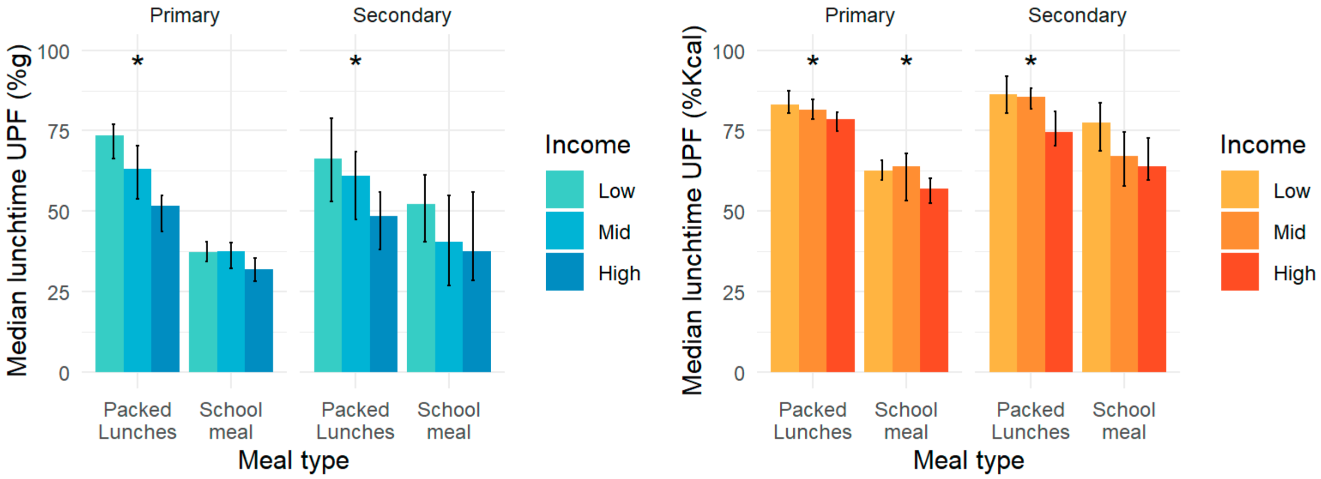

Supplementary Figure S1 Median intake of UPF by school meals stratified by income and school phase.

Note: The left figure presents UPF as %g and right figure as %kcal. * Significant difference across income groups was determined through a Kruskal–Wallis test

Supplementary Table S3 - Logistic regression of the likelihood of consuming minimally and ultra-processed food groups by meal type and school phase (School meals vs reference group packed lunches)

	Primary		Secondary		Total	
**Variable**	**AOR (95% CI)**	**P**	**AOR (95% CI)**	**P**	**AOR (95% CI)**	**P**
**Minimally processed (NOVA1)**						
Drinks	1.9 (1.5,2.3)	<0.01	1.4 (1.1,1.9)	0.02	1.6 (1.3,1.9)	<0.01
Fruit and veg	2.1 (1.6,2.8)	<0.01	0.8 (0.6,1.1)	0.11	1.2 (1.0,1.4)	0.13
Dairy and eggs	2.6 (1.8,3.7)	<0.01	1.7 (1.0,3.0)	0.05	2.2 (1.6,2.9)	<0.01
Starchy foods and legumes	12.5 (9.4,16.7)	<0.01	3.5 (2.5,5.0)	<0.01	6.9 (5.5,8.6)	<0.01
Meat and fish	10.4 (7.7,14.2)	<0.01	3.0 (2.2,4.2)	<0.01	5.6 (4.5,7.0)	<0.01
**Ultra-processed (NOVA4)**						
Processed bread	0.1 (0.1,0.1)	<0.01	0.4 (0.3,0.5)	<0.01	0.2 (0.2,0.2)	<0.01
Sweet and salty snacks	0.2 (0.2,0.2)	<0.01	0.3 (0.2,0.4)	<0.01	0.2 (0.2,0.3)	<0.01
Drinks	0.1 (0.1,0.2)	<0.01	0.8 (0.6,1.1)	0.11	0.3 (0.3,0.4)	<0.01
Condiments	0.5 (0.4,0.6)	<0.01	0.8 (0.6,1.0)	0.06	0.6 (0.5,0.7)	<0.01
Puddings	3.4 (2.6,4.4)	<0.01	1.8 (1.2,2.5)	<0.01	2.5 (2.1,3.1)	<0.01
Ready-to-eat foods	1.4 (1.1,1.9)	0.01	0.8 (0.6,1.1)	0.26	1.1 (0.9,1.3)	0.34
Meat and fish	0.9 (0.7,1.1)	0.28	0.8 (0.6,1.0)	0.09	0.8 (0.7,1.0)	0.02
Vegetables	9.2 (5.4,15.4)	<0.01	4.8 (2.4,9.7)	<0.01	7.1 (4.6,10.8)	<0.01
Cheese	0.2 (0.1,0.4)	<0.01	0.1 (0.0,0.6)	0.01	0.2 (0.1,0.3)	<0.01
Fast foods	11.9 (8.5,16.7)	<0.01	5.4 (3.6,8.0)	<0.01	8.2 (6.4,10.4)	<0.01
Yoghurt and milk	0.2 (0.2,0.3)	<0.01	0.4 (0.3,0.7)	<0.01	0.3 (0.2,0.3)	<0.01

Note: Models adjusted for age, sex, survey year, ethnicity, region, household income and IMD; Packed lunches were reference group

AOR - Adjusted odds ratio; CI - confidence interval

Supplementary Table S4 - Quantile (median) regression on ultra-processed food with an interaction between meal type and income group, stratified by school phase

	Primary				Secondary			
	Model 1[1]		Model 2[2]		Model 1[1]		Model 2[2]	
**Variable**	**Coef (95% CI)**	**P-value**	**Coef (95% CI)**	**P-value**	**Coef (95% CI)**	**P-value**	**Coef (95% CI)**	**P-value**
**UPF (% g)**								
**Intercept**	74.79 (61.55,81.57)	<0.01	69.11 (58.65,83.11)	<0.01	90.38 (57.25,112.44)	<0.01	84.15 (55.95,106.53)	<0.01
**Meal type**								
School meals	-36.82 (-41.35,-30.26)	<0.01	-33.05 (-38.53,-26.89)	<0.01	-12.88 (-29.54,0.66)	0.12	-9.68 (-25.17,-2.25)	0.1
**Income**								
Mid income	-11.24 (-18.25,-1.19)	0.02	-9.19 (-16.61,-3.4)	0.02	-6.82 (-19.63,6.48)	0.39	-6.14 (-18.86,3.94)	0.33
High income	-22.5 (-29.68,-16.05)	<0.01	-20.63 (-26.85,-14.5)	<0.01	-17.98 (-31.8,-3.36)	0.02	-16.08 (-26,-2.96)	<0.01
**Interaction**								
Mid income * School Meal	11.68 (2.06,24.22)	0.03	7.98 (-0.94,17.6)	0.08	-4.96 (-22.49,11.72)	0.65	-3.84 (-23.04,13.24)	0.68
High income * School Meal	17.01 (10.72,24.22)	<0.01	15.6 (9.26,24.7)	<0.01	1.18 (-18.94,17.07)	0.91	-2.47 (-15.65,9.17)	0.77
**UPF (% kcal)**								
**Intercept**	79.98 (75.08,84.49)	<0.01	80.72 (73.09,86.31)	<0.01	84.25 (70.8,102.55)	<0.01	81.36 (64.94,100.41)	<0.01
**Meal type**								
School meals	-21.12 (-24.81,-17.66)	<0.01	-19.92 (-23.73,-15.76)	<0.01	-7.78 (-15.63,-2.38)	0.04	-7.31 (-12.92,2.15)	0.08
**Income**								
Mid income	-1.66 (-5.8,1.25)	0.51	-1.91 (-5.14,0.87)	0.22	-0.2 (-4.57,3.94)	0.95	0.62 (-5.46,6.15)	0.79
High income	-5.72 (-9.65,-2.84)	0.02	-4.56 (-7.64,-2.16)	<0.01	-9.94 (-15.73,-3.24)	<0.01	-8.71 (-13.13,-2.27)	<0.01
**Interaction**								
Mid income * School Meal	3.06 (-2.19,8.09)	0.48	0.99 (-5.87,6.89)	0.76	-3.62 (-7.52,-0.24)	0.09	-8.71 (-21,-0.14)	0.12
High income * School Meal	0.8 (-3.86,6.29)	0.81	-0.12 (-6.73,4.74)	0.96	-8.63 (-18.5,-1.37)	0.11	-4.83 (-13.41,4.78)	0.39

[1]Interaction model with minimal adjustments - age and sex; [2]Interaction model with full adjustments - age, sex, survey year, ethnicity, region, IMD, and income;

Supplementary Table S5 - Sensitivity analysis (1) Additional adjustments for energy, lunch portion and BMI to the quantile regression on ultra-processed food and meal type.

	Primary								Secondary			
	Main analysis[1]		+ Energy (Lunch)[2]		+ Grams (lunch)[3]		+ BMI[4]		Main analysis1		+ Energy (Lun	
**Variable**	**Coef (95% CI)**	**P**	**Coef (95% CI)**	**P**	**Coef (95% CI)**	**P**	**Coef (95% CI)**	**P**	**Coef (95% CI)**	**P**	**Coef (95% CI)**	**P**
**UPF (% g)**												
Packed lunches	(ref)		(ref)		(ref)		(ref)		(ref)		(ref)	
School meals	-24.78 (-28.12,-22.3)	<0.001	-24.32 (-27.64,-21.03)	<0.001	-24.55 (-28.12,-20.09)	<0.001	-24.92 (-28.26,-22.14)	<0.001	-11.64 (-21.03,-6.51)	<0.001	-12.64 (-19.86,-4.82)	<
**UPF (% kcal)**												
Packed lunches	(ref)		(ref)		(ref)		(ref)		(ref)		(ref)	
School meals	-19.64 (-22.26,-17.48)	<0.001	-19.56 (-22.21,-17.28)	<0.001	-19.43 (-22.15,-17.25)	<0.001	-20.04 (-22.47,-17.83)	<0.001	-11.05 (-15.99,-6.96)	<0.001	-10.71 (-14.89,-7.54)	<

[1]Main analysis - adjusted for age, sex, ethnicity, survey year, region, IMD, and income; [2]Additionally adjusted for lunchtime energy intake (grams); [4]Additionally adjusted for BMI

Supplementary Table S6 - Sensitivity analysis (2) Quantile regression on ultra-processed food and meal type with exclusion of sample who meal type was not recorded in the dietary diary

	Primary				Secondary			
	Full-sample (n=1,895)[1]		Reduced sample (n=1,723)[2]		Full-sample (n=3,303)[1]		Reduced sample (n=1,723)[2]	
**Variable**	**Coef (95% CI)**	**P-value**	**Coef (95% CI)**	**P-value**	**Coef (95% CI)**	**P-value**	**Coef (95% CI)**	**P-value**
**UPF (% g)**								
Packed lunches	(ref)		(ref)		(ref)		(ref)	
School meals	-25.55 (-28.41,-21.04)	<0.001	-29.51 (-33.66,-26.98)	<0.001	-12.83 (-20.82,-6.64)	<0.001	-9.72 (-24.99,-4.03)	0.04
**UPF (% kcal)**								
Packed lunches	(ref)		(ref)		(ref)		(ref)	
School meals	-19.89 (-22.41,-17.57)	<0.001	-23.04 (-25.82,-20.54)	<0.001	-12.38 (-16,-8.92)	<0.001	-17.85 (-24.44,-11.08)	<0.001

[1]Fully adjusted model - age, sex, ethnicity, survey year, region, IMD, and income; [2] Analysis run on reduced sample who recorded meal type in the dietary diary (N=1,723) and fully adjusted

Supplementary Table S7 - Sensitivity analysis (3) Quantile regression on ultra-processed food and meal type with exclusion of participants who were estimated to have misreported their energy intake

	Primary				Secondary			
	**Full-sample (n=1,895)[1]**		**Reduced sample (n=1,745)[2]**		**Full-sample (n=1,408)[1]**		**Reduced sample (n=1,073)[2]**	
**Variable**	**Coef (95% CI)**	**P-value**	**Coef (95% CI)**	**P-value**	**Coef (95% CI)**	**P-value**	**Coef (95% CI)**	**P-value**
**UPF (% g)**								
Packed lunches	(ref)		(ref)		(ref)		(ref)	
School meals	-25.55 (-28.41,-21.04)	<0.001	-25.83 (-28.54,-20.98)	<0.001	-12.83 (-20.82,-6.64)	<0.001	-12.71 (-22.45,-4.42)	<0.001
**UPF (% kcal)**								
Packed lunches	(ref)		(ref)		(ref)		(ref)	
School meals	-19.89 (-22.41,-17.57)	<0.001	-20.36 (-22.44,-17.57)	<0.001	-12.38 (-16,-8.92)	<0.001	-10.73 (-15.53,-7.17)	<0.001

[1]Fully adjusted model - age, sex, ethnicity, survey year, region, IMD, and income; [2] Analysis run on reduced sample, excluding 485 participants who were estimated to have misreported their energy intake using the Golberg methods adjusted for children.
